# Early sexual debut is associated with drug use and decreased educational attainment among males and females in Kisumu County, Kenya

**DOI:** 10.1186/s12978-023-01639-3

**Published:** 2023-07-27

**Authors:** Valentine Sing’oei, John K. Owuoth, June Otieno, Adam Yates, Ben Andagalu, Hunter J. Smith, Nathanial K. Copeland, Christina S. Polyak, Trevor A. Crowell, the RV393 Study Team, Rachel Adongo, Rachel Aguttu, Hosea Akala, Julie Ake, Michael Bondo, Erica Broach, Christine Busisa, Jessica Cowden, Mark de Souza, Leigh Anne Eller, Milicent Gogo, Zebiba Hassen, Dale Hu, Michelle Imbach, Anne Juma, Oscar Kasera, Qun Li, Margaret Mbuchi, Mark Milazzo, Kayvon Modjarrad, Eric Ngonda, Chiaka Nwoga, Jacob Nyariro, Jew Ochola, Roseline Ohore, Thomas Okumu, Mary Omondi, Timothy Omondi, Linnah Ooro, Beatrice Orando, Victorine Owira, Roselyn Oyugi, Merlin Robb, Eric Rono, Chi Tran

**Affiliations:** 1 HJF Medical Research International, Ole Odume Road, P.O. Box 37758-00100, Nairobi, Kenya; 2 U.S. Army Medical Research Directorate, P.O. Box 54-40100, Kisumu, Kenya; 3 U.S. Military HIV Research Program, Walter Reed Army Institute of Research, 503 Robert Grant Ave, Silver Spring, MD 20910 USA; 4 Henry M. Jackson Foundation for the Advancement of Military Medicine, Inc., 6720-A Rockledge Drive, Suite 100, Bethesda, MD 20817 USA

**Keywords:** Early sexual debut, HIV, Sex initiation, Sexual behavior, Kenya, Africa South of the Sahara

## Abstract

Differing global sociocultural contexts of sexual relationships influence age at first sexual intercourse with potentially long-lasting region-specific effects such as increased risk of contracting HIV and other sexually transmitted infections (STIs). In these cross-sectional analyses of data from the screening and enrollment visits for an HIV incidence study in Kisumu County, Kenya, we evaluated factors associated with having experienced an early sexual debut (ESD) among males and females aged 18–35 years. Clinical evaluation was performed and sexual behaviors were assessed via questionnaire. ESD was defined as self-reported age 15 years or younger at first sexual intercourse. Robust Poisson regression was used to estimate prevalence ratios (PRs) and 95% confidence intervals (95% CIs) for factors associated with ESD. Of 1057 participants, 542 (51.3%) were female. Participants' median age at study screening was 25 years (interquartile range [IQR]: 22–29), and at sexual debut was 16 years (IQR: 14–17). Five hundred and four participants (47.7%) reported ESD. ESD was less common among females (PR 0.78, CI 0.67–0.90) and participants with more than primary education (PR 0.56, CI 0.47–0.66). ESD was more common in participants with a history of drug use (PR 1.28, CI 1.10–1.49). Drug use removed the protective effect of education (some secondary education or less, no drug use: PR 0.72, CI 0.61–0.85; some secondary education or less, drug use: PR 0.94, CI 0.74–1.18). ESD was common in our study and associated with lower educational attainment and increased likelihood of drug use. Interventions are needed early in life, well before 15 years of age, to encourage engagement in schooling and prevent drug use. Comprehensive sexual education and interventions to prevent drug use may be beneficial before the age of 15 years.

## Plain language summary

Early sexual debut can be defined as first sexual intercourse at or before 15 years of age. There are many social and cultural factors that influence the age of sexual debut. People who start having sex early in life may exhibit behaviors that increase risk for HIV and other sexually transmitted infections. We conducted a study of men and women aged 18–35 years in Kisumu County, Kenya, which included documentation of medical history, physical examination, laboratory tests, and a questionnaire to assess sexual behaviors. Among the 1057 people studied, the average age of sexual debut was 16.0 years for females and 15.4 years for males. A total of 504 (47.7%) participants reported early sexual debut. The data showed that early sexual debut was less common in females and in participants with more years of education. Early sexual debut was more common in participants with a history of drug use. The findings suggest that interventions to prevent early sexual debut might be improved if they focus on educational attainment and prevention of drug use.

## Background

HIV incidence in young populations is strongly influenced by the age of sexual debut [1], which corresponds to the beginning of their risk for sexually-acquired HIV. Early sexual debut (ESD), which is typically defined as first sexual intercourse at or before the age of 15 years, has been linked to key public health threats such as increased likelihood of multiple sexual partnerships in adulthood [1–14], teenage pregnancies [2] and increased risk of HIV and other sexually transmitted infections (STIs) [15, 16]. Of particular concern is the rising number of new HIV diagnoses among adolescents aged 15–24 years, which accounted for 33% of the 52,767 new HIV diagnoses in Kenya in 2017 [17] and the high number of teenage pregnancies; over 13,000 Kenyan girls drop out of school annually due to teenage pregnancy [15].

Another key concern with ESD is that sexual acts at an early age are more likely to be involuntary, forced, coerced, or unplanned and are therefore more likely to occur without the use of condoms [18, 19] or other modes of contraception, which increases the risk of HIV/STIs and unplanned pregnancies [20–22]. Teenage mothers are more likely to experience postpartum depression, drop out of school, and live in poverty compared to those who become pregnant after age 19, thus increasing their chance of engaging in transactional sex and other activities that may increase their risk for HIV and other STIs [23]. The effects of forced sex on mental health may similarly lead to downstream patterns of risky sexual behavior [1, 18]. Young girls who have experienced ESD are also at increased risk of human papillomavirus (HPV) infection due to the rapid physiologic changes of cervical epithelium and immature immune responses [8, 11, 24].

Despite the steady decline of HIV prevalence in Kenya from 5.6% in 2012 [25] to 4.9% in 2017 [17], it is important to note that some regions in Western Kenya continue to suffer from a high HIV burden with almost three times the national prevalence [17]. According to the Kenya Demographic Health Survey (KDHS), 21% of women and 27% of men in Western Kenya, inclusive of Kisumu County, reported sexual intercourse before the age of 15 years [17]. A previous study showed that youth in Western Kenya were likely to initiate sexual activity earlier compared to other regions in Kenya [26].

Repeated population-based behavioral surveys in Uganda showed a 40% decline in HIV seroprevalence among pregnant women attending antenatal clinics and this decrease was attributed to behavior changes observed during that period such as a two-year delay in onset of sexual activity among youth aged 15–24 years [27]. A study from the US showed that, among 18-year-old participants, a history of ESD doubled the odds of having an STI [14]. Similarly, a study done in Nepal showed that those who had ESD were significantly more likely to report history of STIs, multiple sexual partners, sexual violence, transactional sex, and teenage pregnancy compared to those who reported sexual debut at a later age [2]. However, some studies have suggested that the adverse effects of ESD in adolescents could either decrease or disappear in adulthood and thus have little or no association with the development of later sexual behaviours [12, 28].

During adolescence, there is rapid growth characterized by appearance of secondary sexual characteristics; reproductive organ maturation; and physiologic, emotional, and behavioral changes that predispose an individual to exploration and experimentation that can include sexual behaviors that confer risk of HIV and other STIs [29, 30]. The pressure to fit in, fear of rejection among peers, low self-esteem, identity crisis, and sociocultural factors are some of the reasons that potentiate the vulnerability in this age group [19].

Therefore, it is critical to understand the factors associated with ESD and behaviors that may occur after sexual debut in different settings. Updated data from sub-Saharan Africa are essential to informing risk stratification tools for individuals vulnerable to HIV and for developing HIV prevention strategies. The aim of these analyses was to characterize the age of first sexual encounter and relationship of ESD with behaviors observed later in life in Western Kenya.

## Methods

### Study design

The study was conducted at the Kombewa Clinical Research Centre in Kombewa, Kisumu County, Kenya, which is a small town near the northeast shore of Lake Victoria. These analyses utilized cross-sectional data from participants who were screened for eligibility to enroll into an observational, prospective cohort study (RV393) to determine HIV incidence and assess the site’s suitability for future HIV prevention trials. From February 2017 through May 2018, RV393 enrolled males and females who were aged 18–35 years; living without HIV; willing to reside within Kisumu for at least 2 years; able to understand Luo, English, or Kiswahili; and sexually active with 2 or more partners in the last 3 months. Fifty participants living with HIV were also enrolled for masking purposes, so that the HIV status of individuals deemed eligible or ineligible for study participation could not be inferred by observers. Screening for study eligibility entailed HIV testing, pregnancy testing, medical history, physical examination, laboratory evaluations, and administration of an HIV risk assessment questionnaire through face-to-face interviews. STI testing was performed after study eligibility was confirmed. After enrollment into the study, participants were followed every 3 months for up to 2 years. These analyses included all participants who reported age of sexual debut on the HIV risk assessment questionnaire, including individuals who were deemed ineligible for enrollment into the prospective RV393 cohort.

### Measures

#### Sociobehavioral measures

Age was calculated from date of birth on the national identification card or birth certificate. Sex assigned at birth, education level, monthly income, occupation, marital status, and history of drug use in the last three months were self-reported.

The face-to-face behavioral interview included the question, “Have you ever had sexual intercourse?” Those who responded yes were then asked, “Age at first intercourse?” which was a continuous variable. Participants were asked how many sexual partners they had in the past 3 months and, if non-zero, to specify if they had any of the following types of sexual partners: spouse, girlfriend/boyfriend, one-night stand, sex worker, relative, employee, work superior, co-worker, forced partner, and/or other. Transactional sex was assessed by asking, “In the past 3 months, did you have sex with someone in exchange for money, goods, gifts, or favors?” with yes, no, and no answer as response options. Secondary sexual partner was defined as “someone you meet once in a while to have sex with.”

#### Laboratory assessments

HIV testing was carried out for all participants in accordance with the Kenyan Ministry of Health serial HIV testing algorithm with finger prick collection of whole blood for rapid testing using the Determine™ Assay (Abbott Laboratories, Matsudo, Japan), with a reactive result confirmed using First Response® (Premier Medical Corporation limited, Mumbai, India). Discordant HIV results were further tested using the fourth-generation Geenius™ HIV 1/2 Confirmatory Assay (Bio-Rad Laboratories, Johannesburg, South Africa). Upon enrollment in the RV393 cohort, urine samples were collected for *Neisseria gonorrhoeae* and *Chlamydia trachomatis* nucleic acid amplification testing using the Aptima Combo-2 (Hologic Corporation, San Diego, CA, USA). Serum samples were screened for syphilis using the Impact™ RPR carbon agglutination test kit (Alere Technologies, Jena, Germany). All testing was conducted according to package inserts.

### Statistical analyses

These cross-sectional analyses included only those participants who responded to the question about age at first sexual intercourse. ESD was defined as age 15 years or younger at first sexual intercourse, based on the median age at first sexual intercourse in Kenya as seen in KDHS and other studies in Kenya and sub-Saharan Africa [15]. Descriptive analyses were used to investigate frequencies and percentages of ESD, sociodemographic characteristics, sexual behaviors, STIs, and drug use. Chi square or Fisher’s exact tests were used to evaluate differences between groups of interest, including groups with and without ESD. Regression analysis was conducted as a complete-case analysis only, resulting in the exclusion of 34 participants (constituting 3% missingness). Prevalence ratios (PRs) and their corresponding 95% confidence intervals (95% CIs) for factors potentially associated with ESD were estimated using univariable and multivariable Poisson regression with robust sandwich estimators [31]. Variables that were significant at the 0.05 level in the univariable analyses were included in the multivariable model. Estimates were evaluated for statistical significance based on 95% CIs with p < 0.05. Additionally, based on information from community site staff and subject matter experts, an interaction analysis was conducted to examine the potential moderating effect of non-injectable drug use on the relationship between education and early sexual debut in the fully-adjusted multivariable model. All analyses were performed using SAS v9.4.

## Results

### Age of sexual debut

The median and mean ages of sexual debut for the total population were 16 years-old (interquartile range [IQR] 14–17) and 15.7 years-old (standard deviation [SD] 2.6 years), respectively. The mean age of sexual debut for females was 16.0 years (SD 2.3 years), while for males it was 15.4 years (SD 2.7 years, p = 0.001).

A total of 504 (47.7%) participants reported ESD with age of sexual debut at 15 years or younger, 526 (49.8%) had their first experience of sexual intercourse between the ages of 16 and 20 years, and the remaining 26 (2.5%) had their sexual debut at age 21 years or older. Among females, 298 (55.0%) participants reported sexual debut at age 16–20 years and 13 (2.4%) at age 21 years or older. Among men, 228 (44.3%) participants reported sexual debut at age 16–20 years and 14 (2.7%) at age 21 years or older.

### Socio-demographic and behavioral participant characteristics 

Out of 1057 participants included in these analyses, 542 (51.2%) were female and 515 (48.8%) were male. The median age at study screening was 25 years (interquartile range [IQR] 22–29 years).

As compared to participants without ESD, participants who reported ESD were more likely to be male (54.2% vs. 43.8%, p = 0.0008), to not have completed primary school (23.6% vs. 11.2%, p < 0.0001), and to report non-injectable drug use (17.5% vs. 8.7%, p < 0.0001; Table 1). Injectable drug use was uncommon in the cohort, reported by only 2 (0.2%) participants.

**Table 1:** Sociodemographic and behavioral characteristics of participants by age of sexual debut

Characteristics		Early sexual debut		
	Total N = 1057 (%)	No n = 553 (%)	Yes n = 504 (%)	p
Sex				
Male	515 (48.7)	242 (43.8)	273 (54.2)	**0.0008**
Female	542 (51.3)	311 (56.2)	231 (45.8)	
Age				
18–24	500 (47.3)	262 (47.4)	238 (47.2)	0.134
25–29	330 (31.2)	176 (31.8)	154 (30.6)	
30–35	227 (21.5)	115 (20.8)	112 (22.2)	
Education				
None/some primary	181 (17.1)	62 (11.2)	119 (23.6)	** < 0.0001**
Primary/some secondary	448 (42.4)	229 (41.4)	219 (43.5)	
Secondary and above	428 (40.5)	262 (47.4)	166 (32.9)	
Occupation				
Bar/restaurant worker	120 (11.4)	74 (13.4)	46 (9.1)	0.147
Fisherman	158 (14.9)	69 (12.5)	89 (17.7)	
Sex worker	206 (19.5)	116 (21.0)	90 (17.9)	
Other	573 (54.2)	294 (53.2)	279 (55.4)	
Number of partners*				
None	30 (2.8)	23 (4.2)	7 (1.4)	0.223
1–2	398 (37.7)	222 (40.1)	176 (34.9)	
3–5	340 (32.2)	161 (29.1)	179 (35.5)	
6–10	123 (11.6)	60 (10.9)	63 (12.5)	
> 10	166 (15.7)	87 (15.7)	79 (15.7)	
Transactional sex*^β^				
No	395 (37.4)	215 (38.9)	180 (35.7)	0.865
Yes	634 (60.0)	318 (57.5)	316 (62.7)	
Missing/Unknown	28 (2.6)	20 (3.6)	8 (1.6)	
Non-injectable drug use				
No	921 (87.1)	505 (91.3)	416 (82.5)	** < 0.0001**
Yes	136 (12.9)	48 (8.7)	88 (17.5)	
HIV status				
Living without HIV	862 (81.6)	453 (81.8)	409 (81.2)	0.497
Living with HIV	195 (18.4)	100 (18.2)	95 (18.8)	

The table shows the distribution of participant sociodemographic characteristics by self-reported early sexual debut (ESD) status. Chi-squared test of statistical independence was used to calculate p-values for comparisons by early sexual debut. Statistically significant p-values (p < 0.05) are shown in bold. ^β^Transactional sex- Had sex in exchange for money, goods or favors. *In the past 3 months, missing numbers due to don’t know and no answer response options

Females tended to report a higher number of sexual partners in the last 3 months than males (median [IQR] 4 [2–15] vs. 3 [2–4], p < 0.001). Notably, 229 (42.2%) female participants reported having 6 or more partners in the prior 3 months while only 60 (11.6%) males reported the same (p < 0.001). HIV prevalence was 18.5% overall and it was higher in females compared to males (26.2% vs.10.3%, p < 0.001). Among the 646 participants who were enrolled and underwent STI testing, there were relatively few cases of prevalent STIs with 46 (7.1%), 13 (2.0%) and 6 (0.9%) cases of chlamydia, gonorrhea, and syphilis, respectively.

### Factors Associated with ESD

The final multivariable model of factors potentially associated with ESD included sex, age, education, number of partners, and non-injectable drug use. Transactional sex and HIV status were evaluated but not significant in unadjusted modelling and therefore not included in the multivariable model. After controlling for other factors, ESD was less common among females as compared to males (PR 0.78 [95% CI 0.67, 0.90]). As compared to participants who did not complete primary school, having had an early sexual debut was least common among participants who had attained secondary and above education (PR 0.56 [95% CI 0.47, 0.66]) and also less common among participants who had attained primary education and some secondary (PR 0.76 [95% CI 0.66, 0.88]). ESD was more common in participants with a history of non-injectable drug use as compared to those without (PR 1.28 [95% CI 1.10, 1.49]). There was no significant association between ESD and transactional sex, multiple partners, STIs, or HIV (see Table 2).

**Table 2:** Prevalence ratios and confidence intervals for factors potentially associated with early sexual debut

Characteristic	Unadjusted prevalence ratio (95% Confidence Interval)	*P*	Adjusted prevalence ratio (95% Confidence Interval)	*P*
Sex				
Male	Ref.			
Female	**0.80 (0.71, 0.91)**	**0.0008**	**0.78 (0.67, 0.90)**	**0.0008**
Age				
18–24	Ref.			
25–29	0.98 (0.85, 1.14)	0.79	0.88 (0.76, 1.02)	0.09
> 30	1.04 (0.88, 1.22)	0.66	0.91 (0.77, 1.08)	0.30
Education				
None/some primary	Ref.			
Primary/some secondary	**0.74 (0.65, 0.86)**	** < 0.0001**	**0.76 (0.66, 0.88)**	**0.0003**
Secondary or more	**0.59 (0.50, 0.69)**	** < 0.0001**	**0.56 (0.47, 0.66)**	** < 0.0001**
Number of partners				
0–1	Ref.			
2–5	**1.43 (1.18, 1.72)**	**0.0002**	1.11 (0.83, 1.49)	0.48
6–10	**1.41 (1.11, 1.80)**	**0.0054**	1.23 (0.89, 1.69)	0.21
> 11	**1.31 (1.04, 1.65)**	**0.023**	1.30 (0.94, 1.81)	0.11
Transactional sex				
No	Ref.			
Yes	1.09 (0.96, 1.25)	0.19	–	–
Non-injectable drug use				
No	Ref.		Ref.	
Yes	**1.43 (1.24, 1.65)**	** < 0.0001**	**1.28 (1.10, 1.49)**	**0.0012**
HIV status				
Living without HIV	Ref			
Living with HIV	1.03 (0.87, 1.20)	0.76	–	–

Table 2 displays the results from unadjusted and fully adjusted robust Poisson models of factors potentially associated with self-reported early sexual debut (ESD). Variables with unadjusted p < 0.05 were included in the fully adjusted model, with the exception of gender and age, which were selected a priori for inclusion in the multivariable model. Statistically significant prevalence ratios, 95% confidence intervals, and p-values (p ≤0.05) are in bold

**Fig. 1:**
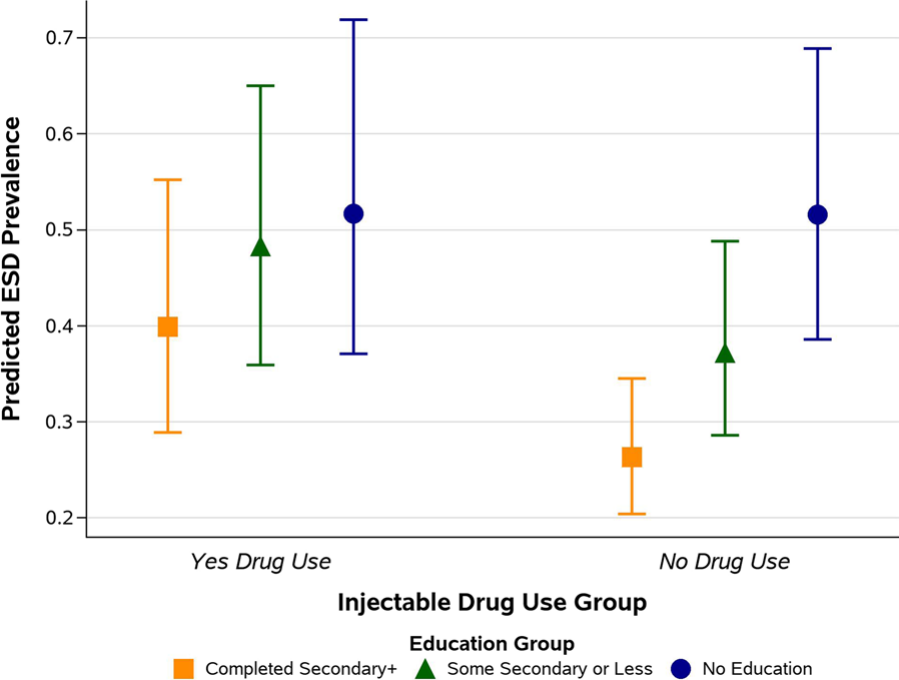
Mediation effect of non-injectable drug use on the association between education and early sexual debut

While a systematic evaluation of potential interactions was not undertaken, site staff and subject matter experts in the Kisumu area suggested that non-injectable drug use and educational status may not be independent. When examining the relationship between ESD, education, and non-injectable drug use, a significant moderating interaction was identified, such that the prevalence of ESD among levels of education was dependent on a participant’s non-injectable drug use status. Among those with no history of non-injectable drug use, higher levels of education were associated with lower risk of ESD; non-injectable drug use removed the protective effect of education (Table 3) (some secondary education or less, no drug use: PR 0.72 [95% CI 0.61, 0.85]; some secondary education or less, drug use: PR 0.94 [95% CI 0.74, 1.18]). By illustrating the predicted ESD prevalence for each combination of non-injectable drug use and education level, Fig. 1 depicts the mediating effect of non-injectable drug use NID use in the past 3 months on the association between highest educational attainment and the predicted prevalence of ESD from the fully adjusted Poisson model.

**Table 3:** Interaction effect between drug use and education as factors associated with early sexual debut

Education level	Non-injectable drug use	Prevalence Ratio (95% Confidence Interval)	P
No education	No	Ref.	
No education	Yes	1.00 (0.76, 1.31)	0.986
Some secondary or less	No	**0.72 (0.61, 0.85)**	**0.0001**
Some secondary or less	Yes	0.94 (0.74, 1.18)	0.577
Completed secondary or more	No	**0.51 (0.42, 0.62)**	** < 0.0001**
Completed secondary or more	Yes	0.77 (0.60, 1.01)	0.055

Table 3 shows fully-adjusted prevalence ratios and 95% confidence intervals from a robust Poisson regression model with self-reported early sexual debut as the outcome. Interaction effects between education and drug use indicate that the association between ESD and education may be mediated by drug use. When drug use was present, there was no independent association between education and ESD. Drug use removed the protective effect of education. Statistically significant prevalence ratios, 95% confidence intervals, and p-values (p ≤ 0.05) are in bold

Figure 1 shows the mean predicted prevalence of having had an early sexual debut by highest educational attainment group within the two levels of non-injectable drug use (Yes-having used NID and No NID use) from the fully adjusted Poisson regression model. Among those who reported using NIDs, there is insufficient evidence to suggest having had an early sexual debut is different by education level. Participants who did not report NID use there is an apparent difference in the predicted prevalence of ESD by education level.

## Discussion

We found that the median age of sexual debut was 16 years old, with almost half of males and females in our study meeting our definition of ESD. This underscores the critical need for sex education and HIV/STI prevention interventions to be made available to vulnerable youth well before this age and the strengthening of systems to retain young people in schools and institutions of higher learning. In 2013, the Kenya Government signed a declaration in which it committed to scaling up comprehensive sexual education in primary schools as part of its strategy to address high rates of teenage pregnancies and to reduce the incidence of HIV among adolescents [32]. However, the sex education program has not achieved optimal implementation due to criticisms and opposition in some areas. Our study suggests a particular need for early sex education in Kisumu County, consistent with previous research where about 36% of women and 16% of men reported their sexual debut by age 12 years [26] and youth in the region have been reported to initiate sex earlier compared to other regions in Kenya [15, 26]. Studies carried out addressing factors associated with ESD in this region suggest early initiation of sex is attributed to a number of factors, including: cultural events such as funerals where community members including adolescents would gather at the deceased home at night for days (providing opportunity for both consensual and non-consensual sex), low socioeconomic status (leading girls to engage in transactional sex), and cultural sleeping arrangements where adolescent boys sleep in their own house (separate from the parents’ house) providing opportunities to invite their girlfriends over for sex [26, 33, 34]. In addition, the main economic activity in this community is fishing which is associated with transactional sex where sex is exchanged for fish thereby increasing the chances of ESD.

In this study, males were more likely to report ESD than females, which is similar to findings from a study that examined outcomes associated with early sexual debut in five sub-Saharan African countries for males and females separately [35] and contrary to the expectation in the African setting where women are considered more prone to ESD due to factors such as early marriages [1, 4, 5, 7, 20, 21, 29, 30, 36, 37]. A survey encompassing 34 countries in sub-Saharan Africa on timing of sexual debut showed that young women typically transition into sexual activity earlier than men [38]. This has been explained by the fact that women develop pubertal changes earlier than boys, and factors such as poverty predispose girls to transactional sex at an early age [39–41]. Studies that show males have a higher likelihood of ESD attribute it to exaggeration of age of sexual debut in face-to-face interviews due to cultural expectations [35, 42]. The changing trend of earlier sexual debut from females to males could also be attributed to increased access to secondary education which typically starts from the age of 14 years, as observed in the KDHS; women with at least secondary education begin sexual activity 3 years later than those with no education [25]. In addition, some studies show that women who have attained a higher level of education are likely to delay sexual initiation [34, 38, 39, 41]. Possible ways in which education favors delay in sexual initiation among young women include instruction on the risks associated with early sex and the development of self-confidence and esteem diminishing the likelihood of coerced sex [41]. These findings support the efforts by governmental and non-governmental organizations in reducing teenage pregnancies by pushing for 100% transition of adolescents from primary to secondary schools and the uptake of sexual education in the academic curriculum.

Our study found an association between ESD and a history of non-injectable drug use, which is consistent with findings from studies performed among adolescents in secondary school [43, 44]. Drug use is prohibited in schools, and children who use drugs may also be engaged in other activities that are socially discouraged or otherwise rebellious, including sexual behaviors that may confer HIV/STI risk. An interaction effect was observed between education and drug use: any amount of education was protective, but drug use gets rid of that protective effect. These findings suggest that interventions addressing ESD may be more successful when they are multi-pronged to not only focus on education attainment but incorporate drug use prevention and management policies.

These analyses yielded important information about sexual debut and associated behaviors that may occur after sexual debut in a large group of male and female participants in an area of sub-Saharan Africa with a high burden of HIV. However, our findings should be interpreted in light of some limitations. First, the study was cross-sectional in nature, so while the temporal sequence of sexual debut and downstream sexual behaviors was clear, temporal relationships between sexual debut and other factors such as drug use and education level were not. Secondly, age at sexual debut and other components of the sexual history were assessed by self-report, with the potential for recall and social desirability biases.

## Conclusion

In conclusion, ESD was reported by almost half of participants in our study in the Kisumu region of Western Kenya, with an average age of sexual debut of around 16 years. ESD was associated with drug use and low educational attainment, which may each predispose to HIV and STI acquisition. Additionally, we observed that drug use eliminated the protective effects resulting from increased education. Prevention programs are likely to be more successful if they are multi-faceted to include environmental, individual, and social context factors in early adulthood besides the strategies of delaying sexual initiation. Lastly, drug use prevention and early and comprehensive sexual education may be beneficial, particularly for young males, as ESD was reported from as young as age 12.

## Data Availability

To request a minimal data set, please contact the data coordinating and analysis center (DCAC) at PubRequest@hivresearch.org and indicate the RV393 study along with the name of the manuscript.

## Abbreviations

CI: Confidence interval
ESD: Early sexual debut
HIV: Human immunodeficiency virus
HPV: Human papillomavirus
IQR: Interquartile range
KEMRI: Kenya Medical Research Institute
KDHS: Kenya Demographic Health Survey
PR: Prevalence ratio
RPR: Rapid plasma reagin
SD: Standard deviation
STI: Sexually transmitted infection

## References

[1] Stöckl H, Kalra N, Jacobi J, Watts C (2013). Is early sexual debut a risk factor for HIV infection among women in Sub-Saharan Africa? A systematic review. Am J Reprod Immunol..

[2] Shrestha R, Karki P, Copenhaver M (2016). Early sexual debut: a risk factor for STIs/HIV acquisition among a nationally representative sample of adults in Nepal. J Community Health.

[3] Udell W, Sandfort T, Reitz E, Bos H, Dekovic M (2010). The relationship between early sexual debut and psychosocial outcomes: a longitudinal study of dutch adolescents. Arch Sex Behav.

[4] Wand H, Ramjee G (2012). The relationship between age of coital debut and HIV seroprevalence among women in Durban, South Africa: a cohort study. BMJ Open.

[5] Yaya S, Bishwajit G (2018). Age at first sexual intercourse and multiple sexual partnerships among women in Nigeria: a cross-sectional analysis. Front Med (Lausanne)..

[6] Sandfort TGM, Orr M, Hirsch JS, Santelli J (2008). Long-term health correlates of timing of sexual debut: results from a national US study. Am J Public Health.

[7] Pettifor AE, Van Der Straten A, Dunbar MS, Shiboski SC, Padian NS (2004). Early age of first sex: a risk factor for HIV infection among women in Zimbabwe. AIDS.

[8] Panatto D, Amicizia D, Trucchi C, Casabona F, Lai PL, Bonanni P (2012). Sexual behaviour and risk factors for the acquisition of human papillomavirus infections in young people in Italy: Suggestions for future vaccination policies. BMC Public Health.

[9] Magnusson BM, Masho SW, Lapane KL (2012). Early age at first intercourse and subsequent gaps in contraceptive use. J Womens Health.

[10] Kim J, Lee JE (2012). Early sexual debut and condom nonuse among adolescents in South Korea. Sex Health.

[11] Houlihan CF, Baisley K, Bravo IG, Kapiga S, de Sanjosé S, Changalucha J (2016). Rapid acquisition of HPV around the time of sexual debut in adolescent girls in Tanzania. Int J Epidemiol.

[12] Heywood W, Patrick K, Smith AMA, Pitts MK (2014). Associations between early first sexual intercourse and later sexual and reproductive outcomes: a systematic review of population-based data. Arch Sex Behav.

[13] Baumann P, Bélanger RE, Akre C, Suris J-C (2011). Increased risks of early sexual initiators : time makes a difference. Sex Health.

[14] Young age at first sexual intercourse and sexually transmitted infections in adolescents and young adults. PubMed-NCBI [Internet]. https://www.ncbi.nlm.nih.gov/pubmed/15800270. Accessed 6 May 2020. 10.1093/aje/kwi095 15800270

[15] National Bureau of Statistics Nairobi K. Republic of Kenya Kenya Demographic and Health Survey 2014. 2015.

[16] Lives T. UNFPA Kenya Annual Report 2017. 2017;

[17] National AIDS and STI Control Programs (NASCOP). Kenya HIV Estimates Report 2018. Kenya HIV Estimates. 2018;1–28.

[18] Moore AM, Awusabo-Asare K, Madise N, John-Langba J, Kumi-Kyereme A (2007). Coerced first sex among adolescent girls in sub-Saharan Africa: prevalence and context. Afr J Reprod Health.

[19] Akintola O, Ngubane L, Makhaba L (2012). “I did it for him, not for me”: An exploratory study of factors influencing sexual debut among female university students in Durban, South Africa. J Health Psychol.

[20] Abma J, Driscoll A, Moore K, Family S, Perspectives P, Feb NJ (2016). Young women ’ s degree of control over first intercourse : an exploratory analysis young women ’ s degree of control over first intercourse : an exploratory analysis. Fam Plann Perspect.

[21] Dickson N, Paul C, Herbison P, Silva P (1998). First sexual intercourse : age, coercion, and later regrets reported by a birth cohort. BMJ.

[22] World Health Organization. Programming for adolescent health and development. Report of a WHO/UNFPA/UNICEF Study Group on Programming for Adolescent Health. World Health Organ Tech Rep Ser. 1999;886:i-vi, 1–260. 10352574

[23] Childbearing NRC (US) P on AP and, Hofferth SL, Hayes CD. Social and economic consequences of teenage childbearing. In: National Academies Press (US); 1987.

[24] Screening in women who have experienced early sexual activity or have been victims of sexual abuse - Clinical Guidelines Wiki [Internet]. https://wiki.cancer.org.au/australia/Clinical_question:Women_experienced_early_sexual_activity_or_victims_of_abuse. Accessed 31 Oct 2019.

[25] National AIDS and STI Control Programme (NASCOP) K. Kenya AIDS indicator survey 2012: final report. Nairobi, NASCOP June 2014. 2014

[26] Tenkorang EY, Maticka-Tyndale E (2014). Individual- and community-level influences on the timing of sexual debut among youth in Nyanza, Kenya. Int Perspect Sex Reprod Health.

[27] Asiimwe-Okiror G, Opio AA, Musinguzi J, Madraa E, Tembo G, Caraël M (1997). Change in sexual behaviour and decline in HIV infection among young pregnant women in urban Uganda. AIDS.

[28] Epsteina M, Manhartb LE, Hilla KG, Baileya JA, David Hawkinsa J, Haggertya KP, Catalano RF (2014). Understanding the link between early sexual initiation and later sexually transmitted infection: Test and replication in two longitudinal studies. J Adolesc Health.

[29] Kassahun EA, Gelagay AA, Muche AA, Dessie AA, Kassie BA (2019). Factors associated with early sexual initiation among preparatory and high school youths in Woldia town, northeast Ethiopia: a cross-sectional study. BMC Public Health.

[30] Peltzer K (2010). Early sexual debut and associated factors among in-school adolescents in eight African countries. Acta Paediatr.

[31] Zou G (2004). A modified poisson regression approach to prospective studies with binary data. Am J Epidemiol.

[32] Bruce ER. education policy analysis archives A peer-reviewed, independent, open access, multilingual journal Committing to Comprehensive Sexuality Education for Young People in Eastern and Southern Africa Independent Researcher and Consultant. 2018;

[33] Manuscript A. Europe PMC Funders Group Disco funerals , a risk situation for HIV infection among youth in. 2009;23 (4):505–9. 10.1097/QAD.0b013e32832605d0 PMC2675523 19165086

[34] Magadi MA, Agwanda AO (2009). Determinants of transitions to first sexual intercourse, marriage and pregnancy among female adolescents: evidence from south nyanza, kenya. J Biosoc Sci.

[35] Seff I, Steiner JJ, Stark L (2021). Early sexual debut: a multi-country, sex-stratified analysis in sub-Saharan Africa. Glob Public Health.

[36] Wall-Wieler E, Roos LL, Nickel NC (2016). Teenage pregnancy: the impact of maternal adolescent childbearing and older sister’s teenage pregnancy on a younger sister. BMC Pregnancy Childbirth.

[37] Walker JA (2012). Women ’ s health and action research centre ( WHARC ) early marriage in Africa—trends, harmful effects and interventions early marriage interventions in africa - trends, harmful effects and. Afr J Reprod Health.

[38] Amo-Adjei J, Tuoyire DA (2018). Timing of sexual debut among unmarried youths aged 15–24 years in Sub-Saharan Africa. J Biosoc Sci.

[39] Marston M, Beguy D, Kabiru C, Cleland J (2013). Predictors of sexual debut among young adolescents in Nairobi’s informal settlements. Int Perspect Sex Reprod Health.

[40] Parcesepe AM, Martin SL, Green S, Mwarogo P, Health C, Hill C (2016). Early sex work initiation and condom use among alcohol-using female sex workers in Mombasa, Kenya: a cross-sectional analysis. Sex Transm Infect.

[41] Okigbo CC, Speizer IS (2015). Determinants of sexual activity and pregnancy among unmarried young women in urban Kenya: a cross-sectional study. PLoS ONE.

[42] Poulin M (2010). Reporting on first sexual experience: The importance of interviewer-respondent interaction. Demogr Res.

[43] Bayissa D, Gebremeskel DM, GutaBayisa MYD (2016). Assessment of early sexual initiation and associated factors among Ambo University undergraduate students, Ambo, Ethiopia. J Health Med Nurs..

[44] Abebe E, Addis A, Asmamaw A, Addisu A, Ayanaw B (2019). Factors associated with early sexual initiation among preparatory and high school youths in Woldia town, northeast Ethiopia : a cross-sectional study. BMC Public Health.

